# Metformin plus L-carnitine enhances brown/beige adipose tissue activity via Nrf2/HO-1 signaling to reduce lipid accumulation and inflammation in murine obesity

**DOI:** 10.1515/med-2024-0900

**Published:** 2024-02-13

**Authors:** Guojin Liang, Jie Fang, Pingping Zhang, Shuxia Ding, Yudan Zhao, Yueying Feng

**Affiliations:** Anesthesiology Department, Ningbo First Hospital, Ningbo, China; Paediatrics Department, Ningbo Women and Children’s Hospital, Zhejiang, 315000, China; Paediatrics Department, Ningbo Women and Children’s Hospital, No. 339 Liuting Street, Ningbo, Zhejiang, 315000, China

**Keywords:** brown adipose tissues, white adipose tissue browning, obesity, inflammation, Nrf2/HO-1 pathway

## Abstract

This study investigated how Metformin (Met) combined with L-carnitine (L-car) modulates brown adipose tissue (BAT) to affect obesity. High-fat-induced obese rats received daily oral gavage with Met and/or L-car, followed by serum biochemical analysis, histopathological observation on adipose tissues, and immunochemistry test for the abdominal expression of BAT-specific uncoupling protein 1 (UCP1). Mouse-embryonic-fibroblast cells were induced into adipocytes, during which Met plus L-car was added with/without saturated fatty acid (SFA). The role of nuclear factor erythroid 2-related factor 2 (Nrf2) in adipocyte browning was investigated by gene silencing. Mitochondria biogenesis in adipocytes was inspected by Mitotracker staining. Nrf2/heme oxygenase-1 (HO-1)/BAT-related genes/proinflammatory marker expressions in adipose tissues and/or adipocytes were analyzed by Western blot, qRT-PCR, and/or immunofluorescence test. Met or L-car improved metabolic disorders, reduced adipocyte vacuolization and swelling, upregulated levels of BAT-related genes including UCP1 and downregulated proinflammatory marker expressions, and activated the Nrf2/HO-1 pathway in adipose tissues of obese rats. Met and L-car functioned more strongly than alone. In adipocytes, Met plus L-car upregulated BAT-related gene levels and protected against SFA-caused inflammation promotion and mitochondria degeneration, which yet was attenuated by Nrf2 silencing. Met plus L-car enhances BAT activity and white adipose tissue browning via the Nrf2/HO-1 pathway to reduce lipid accumulation and inflammation in obese rats.

## 1 Introduction

Obesity is featured as massive fat accumulation that not only causes weight gain but also induces chronic low-grade inflammation, which further triggers vascular dysfunction and metabolic abnormalities and ultimately results in cardiovascular diseases and type 2 diabetes (T2D) [1,2]. These obesity-caused consequences are attributed to excessive expansion and improper remodeling of adipose tissues [1].

There are several types of adipose tissues, such as white and brown adipose tissues (WAT/BAT), all of which are known to participate in energy homeostasis and metabolic modification [3]. BAT combusts lipids through the activation of mitochondrial localized uncoupling protein 1 (UCP1) to transport protons and limit adenosine triphosphate (ATP) production, thus generating heat to contribute to thermogenesis and energy expenditure. WAT mainly functions in energy storage but relates to numerous deleterious effects, including inflammation and mitochondrial dysfunction when unhealthily expanded [4,5]. Derived from precursors of white adipocytes, beige adipose tissues emerge as an intermediate between BAT and WAT and share certain vital functions with BAT [6,7]. The transdifferentiation of white adipocytes into beige adipocytes is called the browning of WAT, which, similar to the increased activity of BAT, confers beneficial outcomes by accelerating intake of glycolipids, reducing insulin secretion requirement, blocking lipid accumulation, suppressing inflammatory response, and improving mitochondrial function [8,9,10].

Metformin (Met) is a biguanide used as the first-line pharmacologic treatment for T2D due to its high safety and effectiveness in lowering glucose levels [11]. One of the mechanisms of Met-regulating metabolic disorders may be the activation of intestinal AMPKα1 to encourage BAT thermogenesis [12]. L-Carnitine (L-car), a biologically active stereoisomer of 3-carboxy-2-hydroxy-*N*,*N*,*N*-trimethyl-1-propanaminium [13], is a constituent of muscle, with a role to transport fatty acids into the mitochondria for oxidation [14]. L-car has been documented to treat insulin resistance (IR) effectively in critically ill acute stroke patients [15] and delay the progression of nonalcoholic fatty liver disease [14]. Notably, L-car promotes brown adipose differentiation and production in goats [16]. These above studies have demonstrated that both Met and L-car facilitate BAT activation and thermogenesis.

Nuclear factor erythroid 2-related factor 2 (Nrf2), a critical modulator of antioxidant signaling, regulates the transcription of various genes coding antioxidant enzymes and cytoprotective proteins [17]. Previously, the activation of the p62/Nrf2/heme oxygenase-1 (HO-1) pathway by ridin has been recorded to promote the effect of brown adipose [18]. Nrf2 directly activates the promoter of UCP1, whereas Nrf2 deficiency compromises the role of UCP1 upregulation in adipocytes [19]. L-car mitigates fructose-mediated lipid accumulation, reactive oxygen species production, and mitochondrial damage, along with increased Nrf2 expression in hepatocytes [20]. Met upregulates Nrf2 expression, thereby abrogating metabolic stress-induced myocardial inflammation and lipid deposition in high-fat diet (HF)-fed mice [21]. L-car works synergistically with Met to improve reproductive performance, IR, and lipid profile in obese women with clomiphene-resistant polycystic ovary syndrome [22]. From these findings, we speculated that the combination of Met and L-car reduces saturated fatty acid (SFA)-induced lipid accumulation and inflammatory responses triggered by regulating BAT activity and WAT browning through the Nrf2/HO-1 pathway.

To test this speculation, an HF-induced obese rat model was established to investigate the effect of Met combined with L-car on BAT activity and browning, and the underlying molecular mechanism was explored with *in vitro* SFA-induced obese models.

## 2 Materials and methods

### 2.1 Ethics statement

The protocol of animal experiments was approved by the Ethics Committee of Zhejiang Baiyue Biotech Co., Ltd., for Experimental Animals Welfare (approval number: ZJBYLA-IACUC-20220901) and carried out under the guidelines of the National Institutes of Health on Animal Care and Use.

### 2.2 Animal experiments

A total of 50 8-week-old male Sprague-Dawley (SD) rats were used in the present research and were housed under specific pathogen-free conditions at 23  ±  1.0 °C, 50  ±  5% humidity, and 12-h dark–light cycle, with free access to water and normal diet. Following a week of acclimatization, all the rats (*n* = 50) were randomly assigned into NF, HF, HF + Met, HF + L-car, and HF + met + L-car groups (*n* = 10 per group). The rats in the NF group were fed with a standard low-fat diet (NF), whereas those in the latter four groups were given an HF (D12451, Research Diets, New Brunswick, NJ, USA; 45 kcal% saturated fat) for 10 weeks [23]. HF-fed rats from the HF + Met and HF + L-car groups received oral gavage of L-car (HY-B0399, C_7_H_15_NO_3_, purity: ≥98.0%, 200 mg/kg, MedChemExpress, Monmouth Junction, NJ, USA) and Met (HY-B0627, C_4_H_11_N_5_, purity: 99.64%, 100 mg/kg, MedChemExpress, USA) once daily, respectively, for 4 weeks [24]. In the HF + met + L-car group, the HF-fed rats were orally administered with L-car (200 mg/kg) and Met (100 mg/kg) in combination once daily by gavage for 4 weeks. At the end of the experiment, all rats underwent anesthetization with 1% pentobarbital sodium (P010, 50 mg/kg; Sigma-Aldrich, St. Louis, MO, USA) after 12-h fasting and then were sacrificed via cervical dislocation. Trunk blood was collected, and serum was obtained via centrifugation at 2,000 × *g* for 20 min and stored at −80°C prior to further use. Abdominal subcutaneous adipose tissues of the rats were dissected, snap-frozen in liquid nitrogen, and stored at −80°C before further use.

### 2.3 Biochemical analysis

The level of fasting blood glucose was measured by a glucose oxidase method-based assay kit (A154-1-1, Jiancheng Bioengineering Institute, Nanjing, China). In brief, serum samples (2.5 µL) were incubated with 250 µL of the working solution containing 4-aminoantipyrine, glucose oxidase, and sodium 3,5-dichloro-2-hydroxybenzenesulfonate at 37°C for 10 min protected from light, followed by the detection of the absorbance at 505 nm with a microplate reader (EMax Plus, Molecular Devices, Sunnyvale, CA, USA). Fasting insulin concentration was determined in rat serum via radioimmunoassay (outsourced by Chemclin Biotechnology Corporation Limited, Beijing, China). Homeostasis model assessment of insulin resistance (HOMA-IR) was calculated based on the formula: HOMA-IR  =  fasting insulin (μU/mL) × fasting blood glucose (mmol/L)/22.5 [23].

### 2.4 Hematoxylin–eosin staining

Rat abdominal subcutaneous adipose tissues were routinely processed and cut into 4 µm-thick slices. After being stained by hematoxylin (HY-N0116, MedChemExpress, USA) and eosin (HY-D0505A, MedChemExpress, USA), the slices were sealed with neutral balsam (N861409, Macklin, China) and subjected to observation via an optical microscope (CX31-LV320, Olympus, Tokyo, Japan) under 100× magnification.

### 2.5 Immunohistochemistry test

Rat abdominal subcutaneous adipose tissue slices were immersed in antigen retrieval solution (P0086, Beyotime, Shanghai, China) and boiled at 95°C for 10 min to repair antigen. Ten-minute treatment with 3% H_2_O_2_ was then conducted to remove endogenous peroxidases in the slices. Later, the slices were blocked for 30 min using 5% bovine serum albumin (BSA; B928042; MACKLIN, China) at 37°C and incubated with primary antibody for UCP1 (ab234430; Abcam, Cambridge, UK) at 4°C overnight. Afterward, secondary antibody HRP-conjugated goat anti-rabbit IgG (31460, ThermoFisher, Waltham, MA, USA) was used to probe the slices for 1 h in the dark, followed by color development using DAB solution (D8001; Sigma-Aldrich, USA). The slices were counterstained with hematoxylin and observed via the optical microscope under 100× magnification.

### 2.6 Western blot

Rat abdominal subcutaneous adipose tissues were subjected to lysis using RIPA Lysis Buffer (20-188; Sigma-Aldrich, USA) to isolate total protein. The protein concentration was then determined with a BCA kit (A53227; ThermoFisher, USA). After that, 30 μg of the isolated protein was separated by SDS–PAGE gel (1615100; BIO-RAD, Hercules, CA, USA) and electrophoretically transferred onto a polyvinylidene fluoride membrane (1620256; BIO-RAD, USA). The blots were blocked in 5% BSA for 1 h at room temperature and probed overnight at 4°C with primary antibodies for Nrf2 (ab92946, 1:1,000; Abcam, UK), HO-1 (ab68477, 1:10,000; Abcam, UK), and glyceraldehyde-3-phosphate dehydrogenase (GAPDH) (ab8245, 1:500; Abcam, UK). Thereafter, 2-h incubation with goat anti-rabbit/mouse IgG secondary antibodies (ab97051/ab6789; Abcam, UK) was conducted at room temperature. Specific proteins were detected using Clarity™ Western ECL Substrate (1705060; BIO-RAD, USA) on an imaging system (LAS-3000; Fujifilm, Tokyo, Japan). Quantitative measurement of immunoreactive band intensities was performed by densitometry analysis using ImageJ software (3.0 version; National Institutes of Health, Bethesda, MA, USA).

### 2.7 Cell culture and transfection

Mouse embryonic fibroblast cell line 3T3-L1 was purchased from Procell (CL-0006; Wuhan, China) and cultured in Dulbecco’s Modified Eagle Medium (DMEM; Procell, China) supplemented with 10% fetal bovine serum (FBS; HY-P2352, MedChemExpress, USA) and 1% penicillin–streptomycin (PB180120; Procell, China) in 5% CO_2_ at 37°C.

Small interfering RNA targeting Nrf2 (SiNrf2; SR427248, OriGene, Rockville, MD, USA) and its negative control (SR30004, OriGene, USA) were separately transfected into 3T3-L1 cells. 3T3-L1 cells were inoculated in 96-well plates at a density of 1 × 10^4^ per well and cultured to become 80% confluent, subsequent to which lipid–RNA complex-based transfection was conducted utilizing Lipofectamine 3000 transfection reagent (L3000015; ThermoFisher, USA). The efficiency of transfection was determined by quantitative reverse transcription-polymerase chain reaction (qRT-PCR).

### 2.8 Cell differentiation and drug treatment

Transfected/non-transfected 3T3-L1 cells were cultured with 1 μM rosiglitazone (ab120762; Abcam, USA) and 10 μg/mL insulin (ab236930; Abcam, USA) in DMEM, with the medium refreshed every 2 days. On day 8, the cells were deemed to differentiate into mature adipocytes and were then harvested [18]. For drug treatment, the adipogenic differentiation culture medium was added with 5 mM L-car [20] 48 h before the cell harvest and/or added with 0.5 mM Met [25] 24 h before the cell harvest.

### 2.9 SFA exposure

Palmitate:stearic mix, referred to as SFA, was prepared by dissolving stearic acid (3.586%):palmitic acid (7.179%) mixture (S4751 and 27734; Sigma-Aldrich, USA) in absolute ethanol, lyophilizing it and reconstituting it in 1 mL 3% BSA. About 2 mM of SFA was used in combination with 5 mM L-car to incubate transfected/non-transfected 3T3-L1 cells in the culture medium 48 h before the 8th day of cell adipogenic differentiation [26], and 0.5 mM Met was also added 24 h prior to the end of the differentiation.

### 2.10 qRT-PCR

Total RNA was extracted from rat adipose tissues, normal 3T3-L1 cells, and transfected/non-transfected adipocytes pretreated with Met plus L-car with or without SFA by employing Trizol reagent (15596026; ThermoFisher, USA). Quantification of the extracted RNA was performed using a spectrophotometer (NanoDrop 2000; ThermoFisher, USA). Then, the RNA was reverse-transcribed utilizing a reverse transcription kit (K1622; Yaanda Biotechnology, Beijing, China) into cDNA, which was later amplified via PCR reaction in a PCR detection system (LightCycler 96; Roche, Indianapolis, IN, USA) equipped with Eastep qPCR Master Mix (LS2062; Promega, Madison, WI, USA). The thermocycler condition was as follows: 95°C for 10 min, followed by 40 circles of 95°C for 15 s and 60°C for 1 min. Relative gene expression values were normalized against the level of GAPDH and were calculated via the 2^−ΔΔCt^ method [27]. The primers used are shown in Table 1.

**Table 1:** Primers used in quantitative reverse transcription polymerase chain reaction for related genes

Genes	Species	Forward (5′–3′)	Reverse (5′–3′)
UCP1	Rat	ACATTGGCGAGAAGGGACAG	GAACTGCAAGGCCCTTTGTG
	Mouse	AGGCTTCCAGTACCATTAGGT	CTGAGTGAGGCAAAGCTGATTT
PRDM16	Rat	CTTGAGGCCTTCCTTGGAGG	GGAAACCGTGAACTGTGCAC
	Mouse	CCACCAGCGAGGACTTCAC	GGAGGACTCTCGTAGCTCGAA
PGC1α	Rat	CTATTCCAGGAGCCAGAGCG	GGGCAGCAGACTACAACAGT
	Mouse	TATGGAGTGACATAGAGTGTGCT	CCACTTCAATCCACCCAGAAAG
IL-6	Rat	ATCTGCCCTTCAGGAACAGC	CTCAATAGCTCCGCCAGAGG
	Mouse	TAGTCCTTCCTACCCCAATTTCC	TTGGTCCTTAGCCACTCCTTC
TNF-α	Rat	AGAGCGGTGATTCAAAGGCA	TTCCACGTCCCATTGGCTAC
	Mouse	CCCTCACACTCAGATCATCTTCT	GCTACGACGTGGGCTACAG
Nrf2	Mouse	CTTTAGTCAGCGACAGAAGGAC	AGGCATCTTGTTTGGGAATGTG
GAPDH	Rat	TGGATAGGGTGGCCGAAGTA	TACAAGGGGAGCAACAGCTG
	Mouse	AGGTCGGTGTGAACGGATTTG	TGTAGACCATGTAGTTGAGGTCA

### 2.11 Immunofluorescence test combined with Mitotracker staining

Adipocyte-differentiating transfected/non-transfected 3T3-L1 cells were treated with Met combined with L-car, together with or without SFA. MitoTracker™ Red CMXRos probes (M7512, 500 nM; ThermoFisher, USA) were employed to track mitochondria in these living cells by being incubated with the cells for 30 min in the darkness. Then, after washing with pre-warmed phosphate-buffered saline (003002; ThermoFisher, USA), the cells were fixed for 15 min in 4% paraformaldehyde and permeabilized using 0.1% Triton X-100 (T8787; Sigma-Aldrich, China) for 10 min. 1% BSA was applied to block the cells for 30 min at 37°C. The cells were then incubated with primary antibody against UCP1 (PA5-120958; ThermoFisher, USA) at 4°C overnight and later with Alexa Fluor™ 488-conjugated goat anti-rabbit IgG secondary antibody (A-11008; ThermoFisher, USA) for 1 h protected from the light. Nucleus staining was performed using 4′,6-diamidino-2-phenylindole (D9542; Sigma-Aldrich, USA). A confocal microscope (Eclipse-Ti; Nikon, Tokyo, Japan) was employed to observe images under 200× magnification.

### 2.12 Statistical analysis

All data from experiments conducted three times were presented as mean ± standard deviation. Statistical analysis was implemented with GraphPad prism (version 8.0; GraphPad Software Inc., San Diego, CA, USA). Kolmogorov–Smirnov’s test and Levene’s test were used to verify the normality of data and the homogeneity of variances, respectively. One-way analysis of variance (ANOVA) was applied to compare mean values among multiple experimental groups, followed by Tukey’s *post hoc* test. Results were considered significant when *P <* 0.05. The animal experiment was carried out in Zhejiang Baiyue Biotech Co.

## 3 Results

### 3.1 Met combined with L-car improved metabolic disorders of obese rats

**Figure 1:**
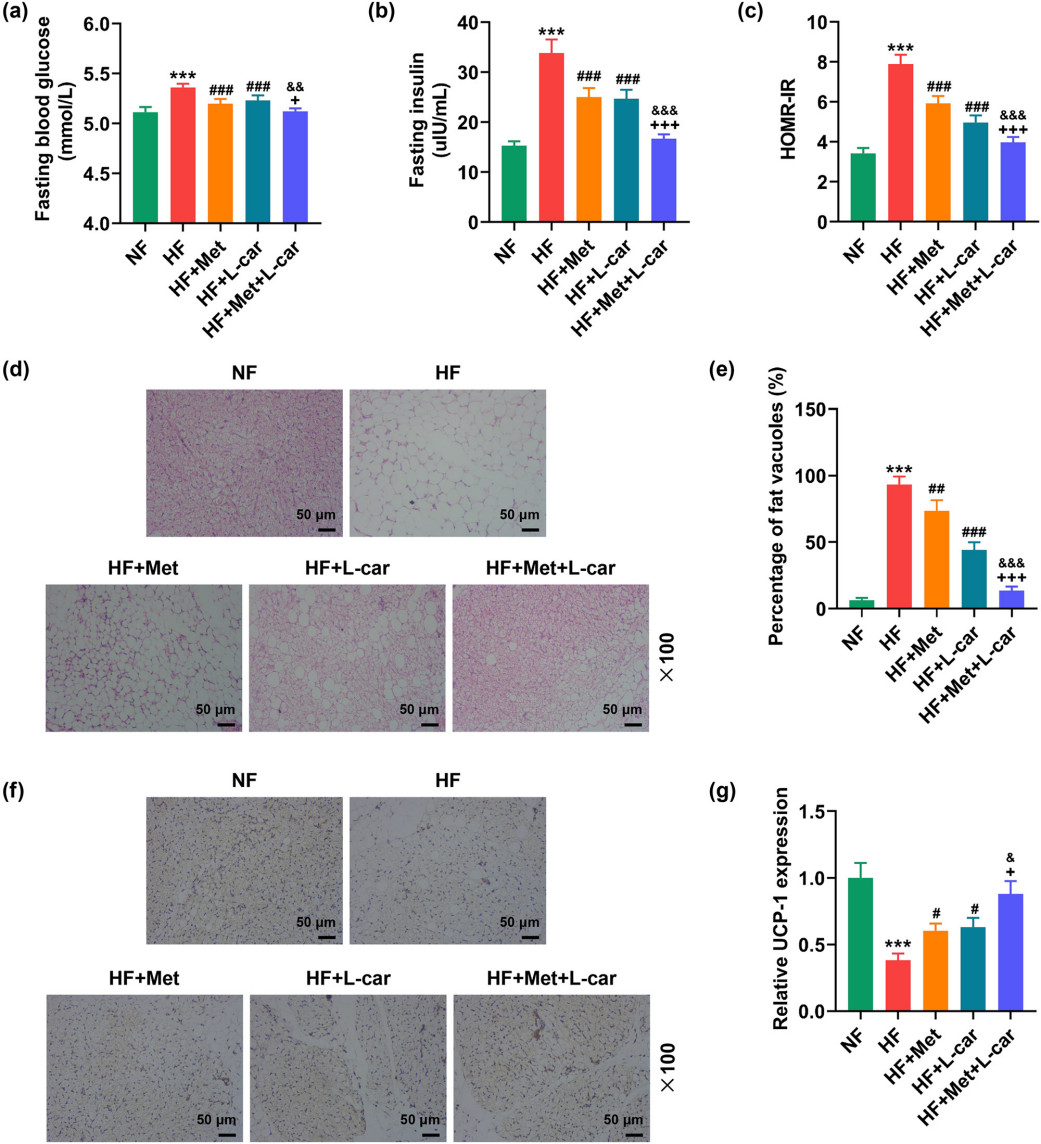
Met combined with L-car improved metabolic disorders, reduced adipocyte vacuolization and swelling and upregulated BAT-specific UCP1 level in abdominal subcutaneous adipose tissues of obese rats. SD rats were fed with normal/high-fat food, and those fed with high-fat food received daily oral gavage of Met and/or L-car. (a) The level of fasting blood glucose was measured by a glucose oxidase method-based assay kit. (b) Fasting insulin concentration was determined via radioimmunoassay. (c) HOMA-IR was calculated. (d) Histological abnormalities in adipose tissue under rat abdominal skin were observed via hematoxylin-eosin staining (magnification, 100×; scale bar, 50 µm). (e) Fat vacuoles of adipose tissue. (f and g) The expression of BAT-specific UCP1 in adipose tissue under rat abdominal skin was detected by the immunochemistry test (magnification, 100×; scale bar, 50 µm). ^+^ *P* < 0.05; ^&&^ *P* < 0.01; ^***^ *P* or ^###^ *P* or ^+++^ *P* or ^&&&^ *P* < 0.001; ^*^ vs NF; ^#^vs HF; ^+^ vs HF + Met; ^&^ vs HF + L-car (NF, normal fat food; HF, high fat food; Met, Metformin; L-car, L-carnitine; HOMA-IR, homeostasis model assessment of insulin resistance; BAT, brown adipose tissues; UCP1, uncoupling protein 1).

Rats in the HF group exhibited elevated levels of fasting blood glucose (Figure 1a, *P* < 0.001), fasting insulin (Figure 1b, *P* < 0.001), and HOMR-IR (Figure 1c, *P* < 0.001). Monotreatment with Met or L-car weakened HF-induced elevation of the above three indexes in rats (Figure 1a–c, *P* < 0.001). The effect of either Met or L-car on decreasing the levels of fasting blood glucose, fasting insulin, and HOMR-IR in HF-fed rats was strengthened after Met and L-car were used in combination (Figure 1a–c, *P* < 0.05).

### 3.2 Met combined with L-car reduced adipocyte vacuolization and swelling and upregulated BAT-specific UCP1 level in abdominal subcutaneous adipose tissues of obese rats

Hemotoxylin–eosin staining results unveiled that HF enlarged vacuoles in adipocytes from the adipose tissues of rats, causing adipocyte swelling (Figure 1d and e, *P* < 0.001). Either Met or L-car alleviated the swelling of adipocytes from the adipose tissues of HF-induced obese rats, with the most obvious alleviation effect induced by combined treatment of Met and L-car (Figure 1d and e, *P* < 0.01). Next, the expression of UCP1 was detected using an immunohistochemistry test, the results of which showed that the abdominal subcutaneous adipose tissues of HF-fed rats had downregulation of BAT-specific UCP1, compared to that of NF-fed rats (Figure 1f and g, *P* < 0.001), while either Met or L-car resisted that reduction of BAT-specific UCP1 expression (Figure 1f and g, *P* < 0.001). Contrasted with Met or L-car monotreatment, the combination of Met and L-car raised BAT-specific UCP1 expression in the abdominal subcutaneous adipose tissues of HF-fed rats more obviously (Figure 1f and g, *P* < 0.001).

### 3.3 Met combined with L-car upregulated BAT-related gene levels, suppressed inflammation, and activated the Nrf2/HO-1 pathway in abdominal subcutaneous adipose tissues of obese rats

**Figure 2:**
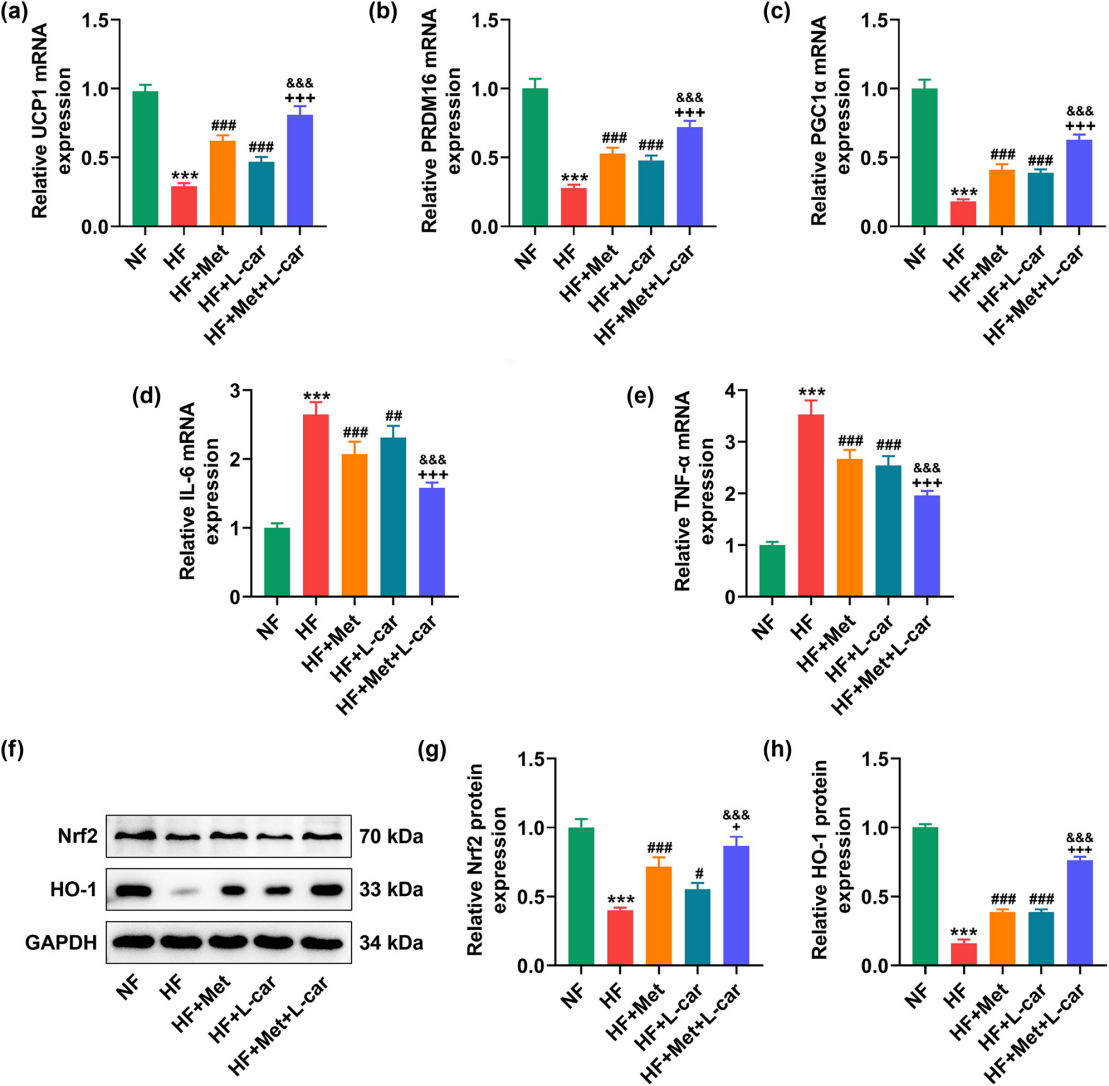
Met combined with L-car upregulated BAT-related gene levels, suppressed inflammation, and activated the Nrf2/HO-1 pathway in abdominal subcutaneous adipose tissues of obese rats. SD rats were fed with normal/high-fat food, and those fed with high-fat food received daily oral gavage with Met and/or L-car. (a–e) The expressions of UCP1, PRDM16, PGC1α, IL-6, and TNF-α in adipose tissue under rat abdominal skin were analyzed by qRT-PCR, with GAPDH used as the normalizer. (f–h). The expressions of Nrf2 and HO-1 in adipose tissue under rat abdominal skin were measured by Western blot, with GAPDH used as the normalizer. ^#^ *P* or ^+^ *P* or ^&^ *P* < 0.05; ^##^ *P* or ^++^ *P* or ^&&^ *P* < 0.01; ^***^ *P* or ^###^ *P* or ^+++^ *P* or ^&&&^ *P* < 0.001; ^*^ vs NF; ^#^ vs HF; ^+^ vs HF + Met; ^&^ vs HF + L-car (NF, normal fat food; HF, high fat food; Met, Metformin; L-car, L-carnitine; BAT, brown adipose tissues; UCP1, uncoupling protein; PRDM16, 1 PR domain containing 16; PGC1α, peroxisome proliferator-activated receptor-gamma co-activator 1-alpha; IL-6, interleukin-6; TNF-α, tumor necrosis factor alpha; Nrf2, nuclear factor (erythroid-derived 2)-like 2; HO-1, heme oxygenase 1; qRT-PCR, quantitative reverse transcription polymerase chain reaction).

Subsequently, the mRNA expressions of brown adipose-related genes, UCP1, PRDM16, and PGC1α, were revealed to be decreased in the abdominal subcutaneous adipose tissues of HF-fed rats through qRT-PCR (Figure 2a–c, *P* < 0.001). However, Met or L-car monotreatment can restore the expressions of these genes in the adipose tissues of HF-fed rats under their abdominal skin (Figure 2a–c, *P* < 0.001), and also the combined treatment of Met and L-car was more effective in increasing expressions of UCP1, PRDM16, and PGC1α in the adipose tissue under their abdominal skin than Met or L-car monotreatment (Figure 2a–c, *P* < 0.001). Moreover, according to qRT-PCR data, in the adipose tissue under rat abdominal skin, HF-induced IL-6 and TNF-α expression levels rose (Figure 2d and e, *P* < 0.001), which was offset by Met or L-car monotreatment (Figure 2d and e, *P* < 0.01). Similarly, a combination of Met and L-car exerted a more potent effect than the monotreatment on decreasing expression levels of these proinflammatory markers (Figure 2d and e, *P* < 0.001). Furthermore, in the adipose tissue under HF-fed rat abdominal skin, decreased expressions of Nrf2 and HO-1 were detected by Western blot (Figure 2d and e, *P* < 0.001), while these HF-caused decreases of Nrf2 and HO-1 expressions were resisted by either Met or L-car (Figure 2f–h, *P* < 0.05). Notably, the effect of Met or L-car on Nrf2 and HO-1 expressions was weaker than that of Met and L-car in combination (Figure 2f–h, *P* < 0.05).

### 3.4 Nrf2 silencing abrogated Met- and L-car-induced upregulation of BAT-related genes in mouse adipocytes

**Figure 3:**
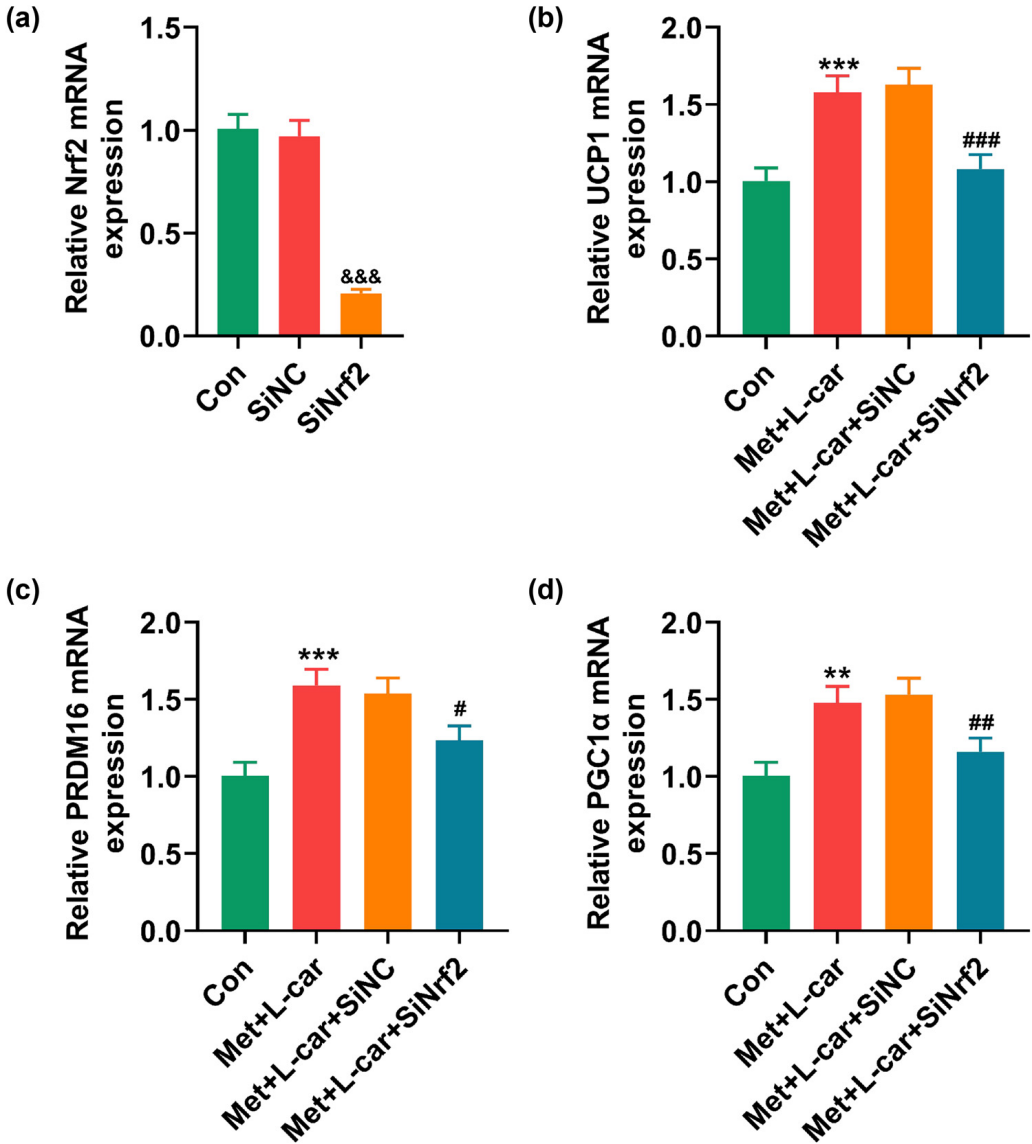
Nrf2 silencing counteracted the combined effect of Met and L-car on upregulation of BAT-related genes in mouse adipocytes. The expression of Nrf2 in SiNrf2/SiNC-transfected 3T3-L1 cells (a) and the expressions of UCP1, PRDM16 and PGC1α (b–d) in SiNrf2/SiNC-transfected 3T3-L1 cells that underwent adipogenic differentiation and treatment with 0.5 mM Met for 24 h and 5 mM L-car for 48 h were analyzed by qRT-PCR, with GAPDH used as the normalizer. ^**^ *P* or ^##^ *P* < 0.01; ^&&&^ *P* or ^***^ *P* or ^###^ *P* < 0.001; ^&^ vs SiNC; ^*^ vs Con; ^#^ vs Met + L-car + SiNrf2 (Met, Metformin; L-car, L-carnitine; BAT, brown adipose tissues; UCP1, uncoupling protein; PRDM16, 1 PR domain containing 16; PGC1α, peroxisome proliferator-activated receptor-gamma co-activator 1-alpha; Nrf2, nuclear factor (erythroid-derived 2)-like 2; SiNrf2, small interfering RNA targeting Nrf2; SiNC, small interfering RNA targeting negative control; qRT-PCR, quantitative reverse transcription polymerase chain reaction).

To decipher the role of Nrf2/HO-1 signaling in Met and L-car-induced browning of adipocytes, 3T3-L1 cells were induced to differentiate into mature adipocytes following Nrf2 silencing. In 3T3-L1 cells, Nrf2 silencing was realized, as evidenced by Figure 3a where Nrf2 expression was diminished after SiNrf2 transfection (Figure 3a, *P* < 0.001). Combined treatment of Met and L-car upregulated UCP1, PRDM16, and PGC1α expressions in adipocytes (Figure 3b–d, *P* < 0.01), the effect of which, however, was discovered to be attenuated by Nrf2 silencing (Figure 3b–d, *P* < 0.01).

### 3.5 Nrf2 silencing weakened the combined effect of Met and L-car on SFA-caused inflammation promotion and mitochondria degeneration in mouse adipocytes

**Figure 4:**
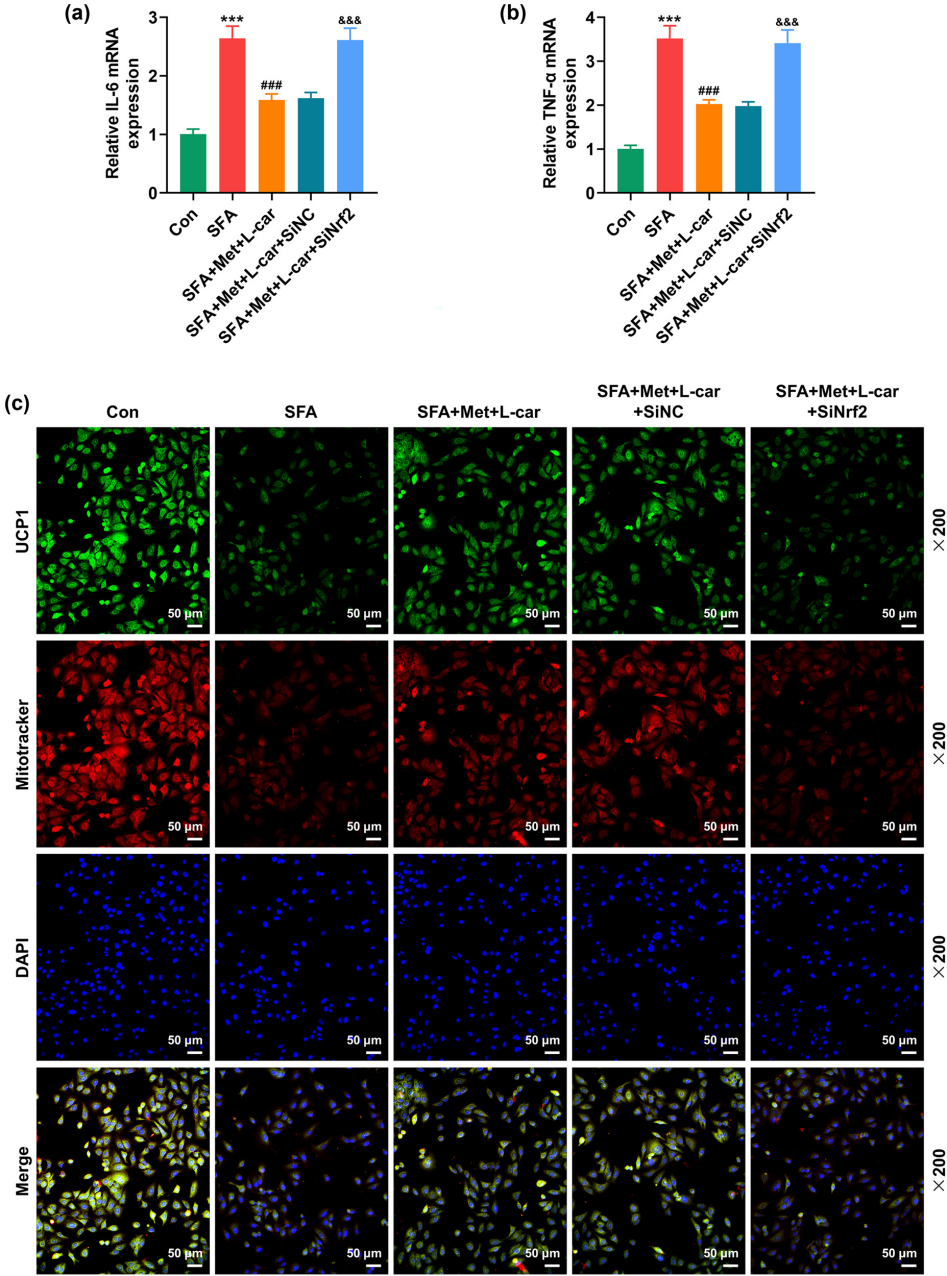
Nrf2 silencing reversed the combined effect of Met and L-car on SFA-caused inflammation promotion and mitochondria degeneration in mouse adipocytes. SiNrf2/SiNC-transfected 3T3-L1 cells underwent adipogenic differentiation and treatment with 5 mM L-car for 48 h in the presence or absence of 2 mM SFA (48 h), accompanied by treatment with 0.5 mM Met for 24 h before cell harvest. (a and b) The expressions of IL-6 and TNF-α in adipocytes were analyzed by qRT-PCR, with GAPDH used as the normalizer. (c) The expression of UCP1 and the activity of mitochondria in adipocytes were detected by immunofluorescence test combined with Mitotracker staining (magnification, × 200; scale bar, 50 µm). ^***^ *P* or ^###^ *P* or ^&&&^ *P* < 0.001; ^*^ vs Con; ^#^ vs SFA; ^&^ vs SFA + Met + L-car + SiNC (Metformin; L-car, L-carnitine; SFA, saturated fatty acid; UCP1, uncoupling protein; IL-6, interleukin-6; TNF-α, tumor necrosis factor alpha; Nrf2, nuclear factor (erythroid-derived 2)-like 2; SiNrf2, small interfering RNA targeting Nrf2; SiNC, small interfering RNA targeting negative control; qRT-PCR, quantitative reverse transcription polymerase chain reaction).

Then, SiNrf2-transfected 3T3-L1 cells undergoing adipogenic differentiation were treated with Met and L-car in combination and exposed to SFA to induce lipid accumulation and initiate inflammatory responses. It was found that IL-6 and TNF-α expressions were boosted by SFA exposure (Figure 4a and b, *P* < 0.001). In SFA-exposed adipocytes, the combination of Met and L-car repressed IL-6 and TNF-α expressions, which was reversed when Nrf2 was silenced (Figure 4a and b, *P* < 0.001). An immunofluorescence test combined with MitoTracker staining confirmed that SFA exposure resulted in the downregulation of UCP1 as well as decreased biologically active mitochondria in adipocytes (Figure 4c). Combined treatment of Met and L-car upregulated UCP1 expression and enhanced the activity of mitochondria in SFA-exposed adipocytes (Figure 4c). Nrf2 silencing almost countervailed the combined effects of Met and L-car on SFA-caused UCP1 downregulation and mitochondria activity suppression in adipocytes (Figure 4c).

## 4 Discussion

Both belonging to adipose tissues, WAT and BAT are two extremely distinct depots with different functions [28]. The browning of WAT converts WAT into beige adipose tissues, which share certain functions with BAT [8,28]. Current research has considered that BAT and the browning of WAT hold the potential to treat metabolic diseases like obesity and T2D [29]. In the present study, we showed that combined treatment of Met and L-car is a promising method for obesity through facilitating the increase of BAT activity and the browning of WAT to reduce SFA-induced lipid accumulation and inflammatory responses.

Met has been used as the first-line therapy with high efficacy to improve glucose metabolism against metabolic diseases [30]. L-car serves as the transport of long-chain fatty acids into mitochondria for their conversion into energy [31], and supplementation with L-car not only reduces the weight of obese patients [32] but also strengthens insulin effect on glycogen storage in T2D patients [33]. In our study, HF-fed obese rats were successfully established, showing increased fasting blood glucose, fasting insulin and HOMR-IR levels, which were later all downregulated by Met or L-car treatment, as anticipated.

Previous studies have demonstrated that Met and L-car can facilitate BAT activities and thermogenesis, and their effects are achieved by virtue of the AMPK pathway activation [12,16]. Hypothalamic AMPK is directly associated with feeding behavior, BAT thermogenesis and also the browning of WAT [34]. BAT, in contrast to WAT, is more mitochondria-enriched and contains smaller fat droplets [29]. BAT, a source of thermogenesis, activates UCP1 to limit ATP production and then oxidizes lipids to generate heat [35,36]. In response to cold exposure, beta-adrenergic stimulation, or exercise, WAT can undergo phenotypic switching (browning) into beige adipose tissues that, though arising from distinct developmental origins from BAT [37], are similar to BAT in morphological features as well as in pro-lipolytic, anti-inflammatory, and UCP1-mediated thermogenic properties [38,39,40,41]. PRDM16 is a transcriptional regulator that induces the expressions of UCP1, PGC1α, and type 2 deiodinase and enhances the uncoupling of respiration, thus maintaining brown fat characteristics, including thermogenic function [42,43]. IL-6 and TNF-α are proinflammatory genes, whose expressions are found to be promoted along with decreased thermogenic activity in obese mice [44]. In this study, both Met and L-car ameliorated adipocyte swelling and fat vacuole enlargement reversed the downregulation of BAT-specific UCP1 and adipose tissue-localized PRDM16, UCP1, and PGC1α and dampened upregulation of IL-6 and TNF-α in the abdominal subcutaneous adipose tissues of obese rats.

Combined treatment of L-car and Met has been demonstrated to improve IR and lipid profile in clomiphene-resistant obese women [22]. This study investigated the efficacy of this combined treatment for obesity and demonstrated that Met and L-car exhibited a stronger effect in combination than they did alone on improving the above-mentioned metabolic disorders, BAT-related gene downregulation, and proinflammatory cytokine upregulation in obese rats.

Mitochondrial respiration is essential to the lipolysis that occurs in BAT or beige adipose tissues to induce adaptive thermogenesis [45,46]. Improvement of mitochondrial biosynthesis contributes to the browning of WAT [47]. Whitening of BAT leads to degeneration of mitochondria [48]. Combined treatment of Met and L-car was also shown to markedly increase active mitochondria in murine adipocytes, suggesting that this combined treatment enhances BAT activity and WAT browning by boosting mitochondrial biosynthesis in rats.

The effect of brown adipose has been recorded to be augmented by the activation of the Nrf2/HO-1 pathway [18]. Nrf2 is a transcription factor that can upregulate the level of cytoprotective enzyme, HO-1 [49], as also shown in our study. Site-specific overexpression of HO-1 diminishes obesity-related proinflammatory cytokine release and lipid accumulation [50]. Notably, Nrf2 also directly induces transcription of UCP1, whose role is compromised in the absence of Nrf2 in adipocytes [19]. L-car attenuates fructose-caused lipid accumulation while upregulating Nrf2 levels in hepatocytes [20]. By increasing Nrf2 expression, Met suppresses metabolic stress-induced myocardial inflammation and lipid accumulation in HF-fed mice [21]. In this study, Nrf2 silencing abrogated Met plus L-car-induced upregulation of PRDM16, UCP1, and PGC1α. It counteracted the combined effect of Met and L-car on mitigating SFA-induced IL-6 and TNF-α expression promotion, UCP1 expression suppression, and mitochondrial degradation in murine adipocytes.

However, our research also has a limitation, which is that we did not accurately screen the high-fat feeding time of mice. Ohtomo et al. found that the expression of UCP1 protein in the BAT of C57BL/6 J mice increased after 2 weeks of high-fat feeding and remained significantly higher than the control group after 4 weeks but almost decreased to the control group level after 20 weeks [51]. After fatty acids enter brown adipocytes, some of them are consumed in the form of heat energy, while the other part accumulates in lipid droplets due to excessive nutrition [51]. Over time, intracellular fat overload, mitochondrial stress, and the whitening process of BATsynergistically damage the ability of BAT to consume fatty acids [51]. Therefore, an HF induces an increase in UCP1 expression in BAT, which is a compensatory adaptation in the early stages of the body. When the compensatory range of the body is exceeded, UCP1 expression decreases, and our future research will further clarify the relationship between high-fat feeding time and UCP1 expression.

In conclusion, the present study provides experimental data which support that combined treatment of Met and L-car enhances BAT activity and WAT browning through activating the Nrf2/HO-1 pathway to reduce lipid accumulation and inflammatory responses in obese murine models.

## References

[1] Koenen M, Hill MA, Cohen P, Sowers JR. Obesity, adipose tissue and vascular dysfunction. Circ Res. 2021;128(7):951–68. 10.1161/circresaha.121.318093. PMC8026272 33793327

[2] Longo M, Zatterale F, Naderi J, Parrillo L, Formisano P, Raciti GA, et al. Adipose tissue dysfunction as determinant of obesity-associated metabolic complications. Int J Mol Sci. 2019;20(9):2358. 10.3390/ijms20092358. PMC6539070 31085992

[3] Frigolet ME, Gutiérrez-Aguilar R. The colors of adipose tissue. Gac Med Mex. 2020;156(2):142–9. 10.24875/gmm.m20000356. 32285854

[4] Cypess AM, Lehman S, Williams G, Tal I, Rodman D, Goldfine AB, et al. Identification and importance of brown adipose tissue in adult humans. N Engl J Med. 2009;360(15):1509–17. 10.1056/NEJMoa0810780. PMC2859951 19357406

[5] Kusminski CM, Bickel PE, Scherer PE. Targeting adipose tissue in the treatment of obesity-associated diabetes. Nat Rev Drug Discov. 2016;15(9):639–60. 10.1038/nrd.2016.75. 27256476

[6] Hocking S, Samocha-Bonet D, Milner KL, Greenfield JR, Chisholm DJ. Adiposity and insulin resistance in humans: The role of the different tissue and cellular lipid depots. Endocr Rev. 2013;34(4):463–500. 10.1210/er.2012-1041. 23550081

[7] Loyd C, Obici S. Brown fat fuel use and regulation of energy homeostasis. Curr Opin Clin Nutr Metab Care. 2014;17(4):368–72. 10.1097/mco.0000000000000063. 24839950

[8] Cheng L, Wang J, Dai H, Duan Y, An Y, Shi L, et al. Brown and beige adipose tissue: A novel therapeutic strategy for obesity and type 2 diabetes mellitus. Adipocyte. 2021;10(1):48–65. 10.1080/21623945.2020.1870060. PMC7801117 33403891

[9] Mu W, Qian S, Song Y, Yang L, Song S, Yang Q, et al. BMP4-mediated browning of perivascular adipose tissue governs an anti-inflammatory program and prevents atherosclerosis. Redox Biol. 2021;43:101979. 10.1016/j.redox.2021.101979. PMC8099561 33895484

[10] Mazibuko-Mbeje SE, Ziqubu K, Dludla PV, Tiano L, Silvestri S, Orlando P, et al. Isoorientin ameliorates lipid accumulation by regulating fat browning in palmitate-exposed 3T3-L1 adipocytes. Metab Open. 2020;6:100037. 10.1016/j.metop.2020.100037. PMC7424791 32812911

[11] Flory J, Lipska K. Metformin in 2019. JAMA. 2019;321(19):1926–7. 10.1001/jama.2019.3805. PMC7552083 31009043

[12] Zhang E, Jin L, Wang Y, Tu J, Zheng R, Ding L, et al. Intestinal AMPK modulation of microbiota mediates crosstalk with brown fat to control thermogenesis. Nat Commun. 2022;13(1):1135. 10.1038/s41467-022-28743-5. PMC8894485 35241650

[13] Agarwal A, Sengupta P, Durairajanayagam D. Role of L-carnitine in female infertility. Reprod Biol Endocrinol. 2018;16(1):5. 10.1186/s12958-018-0323-4. PMC5785901 29373970

[14] Savic D, Hodson L, Neubauer S, Pavlides M. The importance of the fatty acid transporter L-carnitine in non-alcoholic fatty liver disease (NAFLD). Nutrients. 2020;12(8):2178. 10.3390/nu12082178. PMC7469009 32708036

[15] Nejati M, Abbasi S, Farsaei S, Shafiee F. L-carnitine supplementation ameliorates insulin resistance in critically ill acute stroke patients: a randomized, double-blinded, placebo-controlled clinical trial. Res Pharm Sci. 2022;17(1):66–77. 10.4103/1735-5362.329927. PMC8621844 34909045

[16] Wang Y, Chen X, Fan W, Zhang X, Zhan S, Zhong T, et al. Integrated application of metabolomics and RNA-seq reveals thermogenic regulation in goat brown adipose tissues. FASEB J: Off Publ Fed Am Soc Exp Biol. 2021;35(9):e21868. 10.1096/fj.202100493RR. 34449920

[17] Xia Y, Zhai X, Qiu Y, Lu X, Jiao Y. The Nrf2 in obesity: A friend or foe? Antioxidants (Basel, Switz). 2022;11(10):2067. 10.3390/antiox11102067. PMC9598341 36290791

[18] Tsai YC, Wang CW, Wen BY, Hsieh PS, Lee YM, Yen MH, et al. Involvement of the p62/Nrf2/HO-1 pathway in the browning effect of irisin in 3T3-L1 adipocytes. Mol Cell Endocrinol. 2020;514:110915. 10.1016/j.mce.2020.110915. 32540261

[19] Chang SH, Jang J, Oh S, Yoon JH, Jo DG, Yun UJ, et al. Nrf2 induces Ucp1 expression in adipocytes in response to β3-AR stimulation and enhances oxygen consumption in high-fat diet-fed obese mice. BMB Rep. 2021;54(8):419–24. 10.5483/BMBRep.2021.54.8.023. PMC8411042 33691909

[20] Montesano A, Senesi P, Vacante F, Mollica G, Benedini S, Mariotti M, et al. L-Carnitine counteracts in vitro fructose-induced hepatic steatosis through targeting oxidative stress markers. J Endocrinol Invest. 2020;43(4):493–503. 10.1007/s40618-019-01134-2. PMC7067714 31705397

[21] Ge CX, Xu MX, Qin YT, Gu TT, Lou DS, Li Q, et al. Endoplasmic reticulum stress-induced iRhom2 up-regulation promotes macrophage-regulated cardiac inflammation and lipid deposition in high fat diet (HFD)-challenged mice: Intervention of fisetin and metformin. Free Radic Biol Med. 2019;141:67–83. 10.1016/j.freeradbiomed.2019.05.031. 31153974

[22] El Sharkwy I, Sharaf El-Din M. l-Carnitine plus metformin in clomiphene-resistant obese PCOS women, reproductive and metabolic effects: A randomized clinical trial. Gynecol Endocrinol: Off J Int Soc Gynecol Endocrinol. 2019;35(8):701–5. 10.1080/09513590.2019.1576622. 30806102

[23] Zhao M, Zang B, Cheng M, Ma Y, Yang Y, Yang N. Differential responses of hepatic endoplasmic reticulum stress and inflammation in diet-induced obese rats with high-fat diet rich in lard oil or soybean oil. PLoS One. 2013;8(11):e78620. 10.1371/journal.pone.0078620. PMC3819370 24223162

[24] Zayed EA, AinShoka AA, El Shazly KA, Abd El Latif HA. Improvement of insulin resistance via increase of GLUT4 and PPARγ in metabolic syndrome-induced rats treated with omega-3 fatty acid or l-carnitine. J Biochem Mol Toxicol. 2018;32(11):e22218. 10.1002/jbt.22218. 30256492

[25] Breining P, Jensen JB, Sundelin EI, Gormsen LC, Jakobsen S, Busk M, et al. Metformin targets brown adipose tissue in vivo and reduces oxygen consumption in vitro. Diabetes Obes Metab. 2018;20(9):2264–73. 10.1111/dom.13362. 29752759

[26] Youssef-Elabd EM, McGee KC, Tripathi G, Aldaghri N, Abdalla MS, Sharada HM, et al. Acute and chronic saturated fatty acid treatment as a key instigator of the TLR-mediated inflammatory response in human adipose tissue, in vitro. J Nutr Biochem. 2012;23(1):39–50. 10.1016/j.jnutbio.2010.11.003. PMC3243902 21414768

[27] Livak KJ, Schmittgen TD. Analysis of relative gene expression data using real-time quantitative PCR and the 2(-Delta Delta C(T)) method. Methods (San Diego, Calif). 2001;25(4):402–8. 10.1006/meth.2001.1262. 11846609

[28] Boucher JM, Ryzhova L, Harrington A, Davis-Knowlton J, Turner JE, Cooper E, et al. Pathological conversion of mouse perivascular adipose tissue by notch activation. Arterioscler Thromb Vasc Biol. 2020;40(9):2227–43. 10.1161/atvbaha.120.314731. PMC7483939 32640901

[29] Jeremic N, Chaturvedi P, Tyagi SC. Browning of white fat: Novel insight into factors, mechanisms, and therapeutics. J Cell Physiol. 2017;232(1):61–8. 10.1002/jcp.25450. PMC6567990 27279601

[30] LaMoia TE, Shulman GI. Cellular and molecular mechanisms of metformin action. Endocr Rev. 2021;42(1):77–96. 10.1210/endrev/bnaa023. PMC7846086 32897388

[31] Bremer J. Carnitine--metabolism and functions. Physiol Rev. 1983;63(4):1420–80. 10.1152/physrev.1983.63.4.1420. 6361812

[32] Askarpour M, Hadi A, Miraghajani M, Symonds ME, Sheikhi A, Ghaedi E. Beneficial effects of l-carnitine supplementation for weight management in overweight and obese adults: An updated systematic review and dose-response meta-analysis of randomized controlled trials. Pharmacol Res. 2020;151:104554. 10.1016/j.phrs.2019.104554. 31743774

[33] Adeva-Andany MM, Calvo-Castro I, Fernández-Fernández C, Donapetry-García C, Pedre-Piñeiro AM. Significance of l-carnitine for human health. IUBMB Life. 2017;69(8):578–94. 10.1002/iub.1646. 28653367

[34] López M. Hypothalamic AMPK and energy balance. Eur J Clin Invest. 2018;48(9):e12996. 10.1111/eci.12996. PMC6175178 29999521

[35] Lidell ME. Brown adipose tissue in human infants. Handb Exp Pharmacol. 2019;251:107–23. 10.1007/164_2018_118. 29675580

[36] Lidell ME, Betz MJ, Enerbäck S. Brown adipose tissue and its therapeutic potential. J Intern Med. 2014;276(4):364–77. 10.1111/joim.12255. 24717051

[37] Rui L. Brown and beige adipose tissues in health and disease. Compr Physiol. 2017;7(4):1281–306. 10.1002/cphy.c170001. PMC6192523 28915325

[38] Kaisanlahti A, Glumoff T. Browning of white fat: Agents and implications for beige adipose tissue to type 2 diabetes. J Physiol Biochem. 2019;75(1):1–10. 10.1007/s13105-018-0658-5. PMC6513802 30506389

[39] Waldén TB, Hansen IR, Timmons JA, Cannon B, Nedergaard J. Recruited vs nonrecruited molecular signatures of brown, “brite,” and white adipose tissues. Am J Physiol Endocrinol Metab. 2012;302(1):E19–31. 10.1152/ajpendo.00249.2011. 21828341

[40] Wang QA, Tao C, Gupta RK, Scherer PE. Tracking adipogenesis during white adipose tissue development, expansion and regeneration. Nat Med. 2013;19(10):1338–44. 10.1038/nm.3324. PMC4075943 23995282

[41] Rosenwald M, Perdikari A, Rülicke T, Wolfrum C. Bi-directional interconversion of brite and white adipocytes. Nat Cell Biol. 2013;15(6):659–67. 10.1038/ncb2740. 23624403

[42] Seale P, Bjork B, Yang W, Kajimura S, Chin S, Kuang S, et al. PRDM16 controls a brown fat/skeletal muscle switch. Nature. 2008;454(7207):961–7. 10.1038/nature07182. PMC2583329 18719582

[43] Seale P, Kajimura S, Yang W, Chin S, Rohas LM, Uldry M, et al. Transcriptional control of brown fat determination by PRDM16. Cell Metab. 2007;6(1):38–54. 10.1016/j.cmet.2007.06.001. PMC2564846 17618855

[44] Martins FF, Bargut TCL, Aguila MB, Mandarim-de-Lacerda CA. Thermogenesis, fatty acid synthesis with oxidation, and inflammation in the brown adipose tissue of ob/ob (-/-) mice. Ann Anat = Anatomischer Anzeiger: Off organ Anatomische Ges. 2017;210:44–51. 10.1016/j.aanat.2016.11.013. 27986616

[45] Wu J, Boström P, Sparks LM, Ye L, Choi JH, Giang AH, et al. Beige adipocytes are a distinct type of thermogenic fat cell in mouse and human. Cell. 2012;150(2):366–76. 10.1016/j.cell.2012.05.016. PMC3402601 22796012

[46] Lowell BB, Spiegelman BM. Towards a molecular understanding of adaptive thermogenesis. Nature. 2000;404(6778):652–60. 10.1038/35007527. 10766252

[47] Park SS, Lee YJ, Kang H, Yang G, Hong EJ, Lim JY, et al. Lactobacillus amylovorus KU4 ameliorates diet-induced obesity in mice by promoting adipose browning through PPARγ signaling. Sci Rep. 2019;9(1):20152. 10.1038/s41598-019-56817-w. PMC6934708 31882939

[48] Kotzbeck P, Giordano A, Mondini E, Murano I, Severi I, Venema W, et al. Brown adipose tissue whitening leads to brown adipocyte death and adipose tissue inflammation. J Lipid Res. 2018;59(5):784–94. 10.1194/jlr.M079665. PMC5928436 29599420

[49] Liu X, Yuan X, Liang G, Zhang S, Zhang G, Qin Y, et al. BRG1 protects the heart from acute myocardial infarction by reducing oxidative damage through the activation of the NRF2/HO1 signaling pathway. Free Radic Biol Med. 2020;160:820–36. 10.1016/j.freeradbiomed.2020.09.012. 32950688

[50] Drummond GS, Baum J, Greenberg M, Lewis D, Abraham NG. HO-1 over expression and underexpression: Clinical implications. Arch Biochem Biophys. 2019;673:108073. 10.1016/j.abb.2019.108073. PMC6748652 31425676

[51] Ohtomo T, Ino K, Miyashita R, Chigira M, Nakamura M, Someya K, et al. Chronic high-fat feeding impairs adaptive induction of mitochondrial fatty acid combustion-associated proteins in brown adipose tissue of mice. Biochem Biophys Rep. 2017;10:32–8. 10.1016/j.bbrep.2017.02.002. PMC5614659 28955734

